# Immune Thrombocytopenia Following COVID-19 Vaccination in a Pediatric Patient

**DOI:** 10.7759/cureus.117319

**Published:** 2026-10-01

**Authors:** Christy M Syriac, John D Petersen, Michael Bolaris, Shannon M Thyne

**Affiliations:** 1 Pediatrics, Olive View-University of California, Los Angeles Medical Center, Sylmar, USA; 2 Pediatrics, University of California, Los Angeles David Geffen School of Medicine, Los Angeles, USA; 3 Pediatrics/Pediatric Infectious Disease, Rancho Los Amigos National Rehabilitation Center, Downey, USA; 4 Pediatrics/Pediatric Infectious Disease, University of California, Los Angeles David Geffen School of Medicine, Los Angeles, USA

**Keywords:** covid-19 vaccination complication, covid-19 vaccine, moderna covid-19 vaccine, pediatric immune thrombocytopenia, vaccine-induced immune thrombocytopenia

## Abstract

Immune thrombocytopenia (ITP) is the most common acquired bleeding disorder in children, with further delineation into primary and secondary causes; it is immune-mediated and typically self-limited. Primary ITP is often seen in children with a history of a preceding infection that has subsequently resolved. Additionally, ITP can be seen in children within six weeks of vaccination with measles-mumps-rubella and varicella, and in adults, ITP has developed following messenger RNA (mRNA) vaccination. We present the case of a three-year-old boy who presented with petechiae and purpura eight days after Moderna COVID-19 vaccination and was found to have severe thrombocytopenia with a platelet count of 3,000/mm³. He was thought to have secondary ITP from COVID-19 vaccination. To date, there have been no published reports of ITP presenting in children following mRNA COVID-19 vaccination. This case brings to attention the possible complication of ITP after COVID-19 vaccination with spontaneous resolution without medical intervention and the need for further clinical monitoring of symptoms after COVID-19 vaccination.

## Introduction

Immune thrombocytopenia (ITP) is the most common acquired bleeding disorder in children and is characterized by isolated thrombocytopenia of <100 × 10^9^/L platelets (normal range for platelets is 150,000-450,000/mm³) [1]. ITP is often seen in children with a history of a preceding viral or acute gastrointestinal infection and presents with signs of mucocutaneous bleeding such as petechiae, purpura, ecchymosis, or epistaxis. ITP has a peak incidence between two and six years of age [2]. Primary ITP is typical in pediatric patients and often benign and self-limited, with spontaneous recovery of platelet counts in three to six months. Secondary ITP can be caused by an underlying autoimmune condition, drugs, malignancy, infections, or immunodeficiency. Vaccination can trigger autoimmunity, with reports of ITP occurring in children after routine vaccination with measles-mumps-rubella (MMR) and varicella, and reports of ITP presenting in adults following messenger RNA (mRNA) vaccination [3]. A causal association between MMR and ITP has been reported with an incidence of 0.087-4 per 100,000 doses seen in children [4]. In May 2025, the U.S. Food and Drug Administration (FDA) approved Moderna’s next-generation mRNA COVID-19 vaccine for children ages six months through eleven years with at least one underlying condition putting them at high risk for severe outcomes from COVID-19. In adults, thrombocytopenia induced by COVID-19 vaccination showed an incidence of 0.80 to 11.3 per million doses [4]. However, there are currently no published reports of ITP presenting in children following mRNA vaccination. In this case, we present the case of a pediatric patient with ITP after receiving their first dose of the Moderna COVID-19 mRNA vaccine. Consent to present this case was obtained from the parents of this patient. This rare occurrence after COVID-19 vaccination suggests that further surveillance is required.

## Case presentation

A three-year-old boy presented to the emergency department with new-onset bruising for two days. The day before presentation, his family had noted bruising on his lower extremities and called his pediatrician, who attributed the bruising to falling off his toddler bed. On the day he presented to the emergency department, the family noted new bruising on his left shoulder and worsening of the initial bruising on his shins. The family again called the pediatrician, who referred them to the emergency department for further evaluation.

In the emergency department, the family denied fevers, epistaxis, gum bleeding, hematuria, bleeding into joints, weight loss, and decreased appetite. The patient had received his first Moderna COVID-19 vaccine eight days before onset of symptoms. He had non-bloody diarrhea for two to three days after receiving his vaccine but denied any recent rhinorrhea and nasal congestion. His only medication was daily Flovent for asthma, and he reported no other medical history. He also reported being fully vaccinated, including against influenza, with none given in the past 11 months. There was no known family history of autoimmune conditions or bleeding disorders. On examination, he was afebrile, with other vital signs within normal limits for age. His physical examination was notable for ecchymosis on his left shoulder (Figure 1), and multiple petechiae, purpura, and ecchymoses were identified on his bilateral shins (Figure 2). Family consented to the photos taken for this case. At this initial presentation, there was no mucosal bleeding noted. The remainder of his physical examination was normal for age.

**Figure 1:**
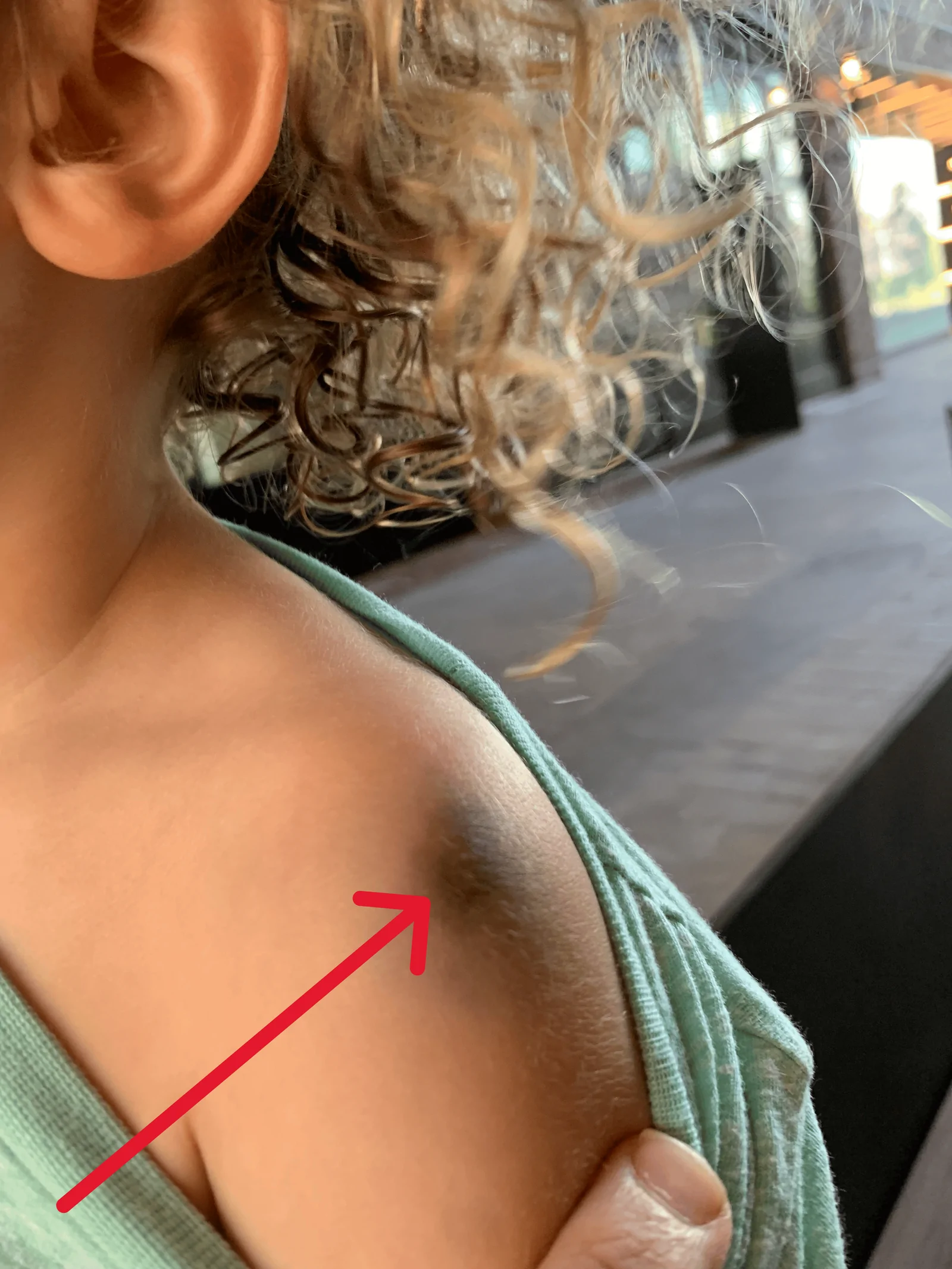
Ecchymosis on the left shoulder.

**Figure 2:**
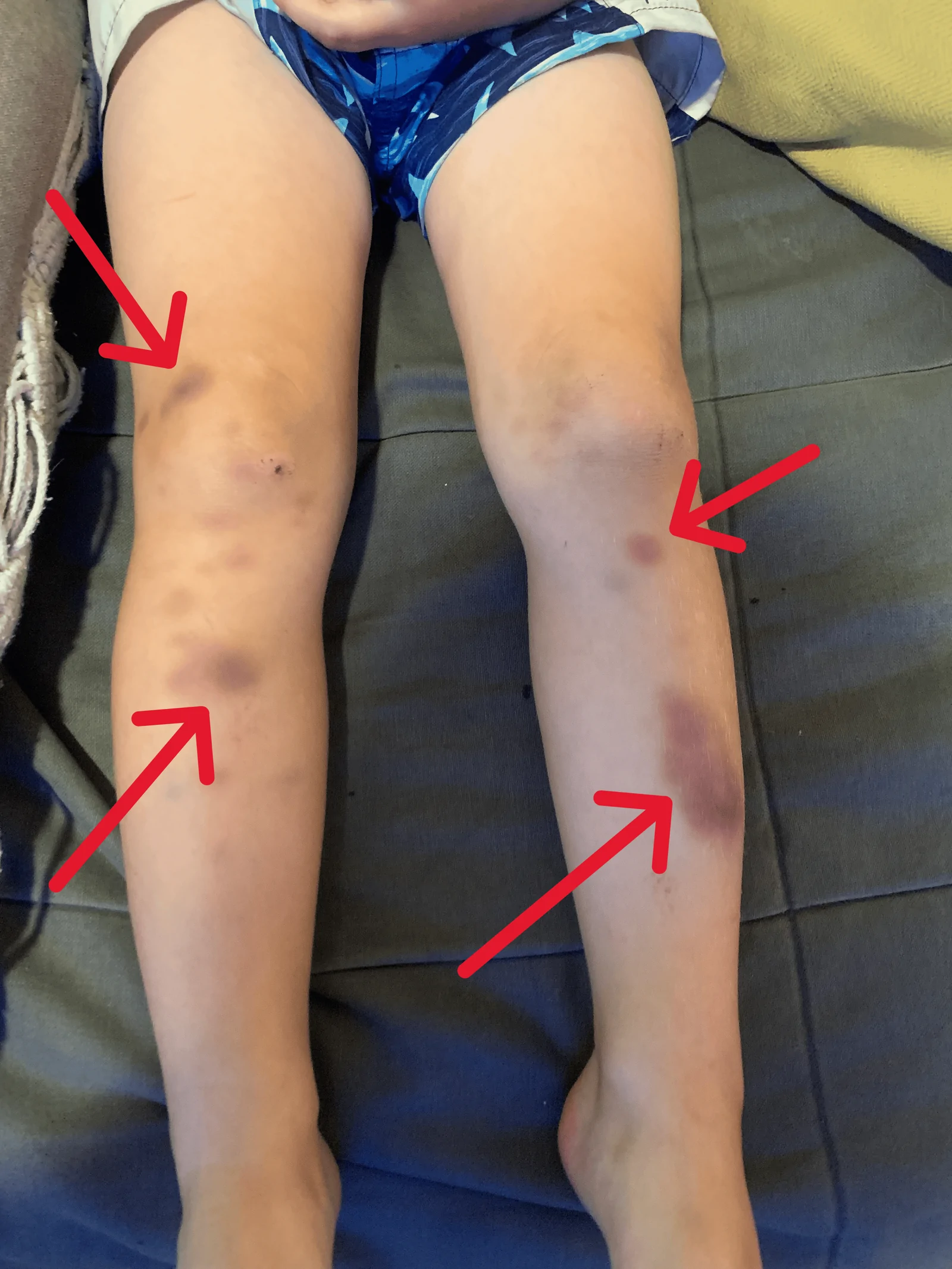
Purpura and ecchymosis on bilateral shins.

Laboratory testing revealed normal basic metabolic panel and liver function testing. Complete blood count demonstrated severe thrombocytopenia (platelets 3,000/mm³) and a normal white blood cell count and hemoglobin (12.8 g/dL). The partial thromboplastin time was 44.2 seconds (reference range: 27-38 seconds), and prothrombin time was 11.2 seconds with an international normalized ratio of 0.9. He tested negative for COVID-19 (Table 1).

**Table 1:** Initial laboratory tests.

Parameters	Reference ranges	Patient value
Hemoglobin	10.1–12.5 g/dL	12.8 g/dL
Platelet count	150,000–450,000/mm³	3,000/mm³
Partial thromboplastin time	27–38 seconds	44.2 seconds
Prothrombin time	11–13.5 seconds	11.2 seconds
International normalized ratio	0.8–1.3	0.9
SARS-CoV-2 real-time reverse-transcription polymerase chain reaction, nasal swab	Negative	Negative

The patient was discharged home with bleeding precautions, and an outpatient follow-up with Pediatric Hematology was scheduled for two days later. On the day after discharge, his family noted slight mucosal bleeding along his gum line. He was seen in Pediatric Hematology clinic with resolution of gum bleeding and no new bruising. A follow-up complete blood count on that day showed continued severe thrombocytopenia (platelets: 5,000/mm³). Immunoglobulin A was 37 mg/dL (reference range: 14-212 mg/dL), and immunoglobulin G was 421 mg/dL (reference range: 485-1,160 mg/dL). The decision was made to defer medical treatment for ITP given no new bleeding and capacity for close outpatient monitoring. Over the next 10 days, ecchymoses resolved and the child remained without new symptoms. His repeat complete blood count showed a platelet count of 28,000/mm³ (Table 2).

**Table 2:** Blood laboratory tests at follow-up visits.

Parameters	Reference ranges	Patient value
Platelet count	150,000–450,000/mm³	5,000/mm³
Platelet count (after resolution of ecchymosis)	150,000–450,000/mm³	28,000/mm³
Immunoglobulin A	14–212 mg/dL	37 mg/dL
Immunoglobulin G	485–1,160 mg/dL	421 mg/dL

## Discussion

ITP is an acquired thrombocytopenia due to antibodies against platelet antigens and is the most common acquired thrombocytopenia in children [1]. Primary ITP is defined as a platelet count <100 × 10^9^/L not due to any specific etiology [1]. Secondary ITP is thrombocytopenia due to an underlying disorder such as a viral illness, autoimmune disorders, or immunodeficiency [1]. Certain vaccines such as MMR, *Haemophilus influenzae* type b, and hepatitis B have been implicated in vaccine-associated ITP, with an incidence of 1% of all ITP cases [3]. Because the incidence of acute ITP in children shows a peak between the ages of two to six years [2], it may become more common to see ITP after vaccination for COVID-19 in the younger population, who are still only recently eligible for vaccination under emergency use authorization.

In a review of 20 case reports, new-onset thrombocytopenia occurred within two weeks of mRNA vaccination, with ages ranging from 22-73 years, and predominantly in females [5]. Causes of thrombocytopenia in these cases include secondary ITP due to mRNA vaccination, primary ITP occurring around the time of vaccine administration, or worsening of undiagnosed ITP [5]. Overall, it remains unclear what would trigger ITP after COVID-19 vaccination, although one theoretical mechanism involves antibodies against a novel antigen, the outer lipid layer of nanoparticles attached to platelets, as there have been antibodies detected post-COVID-19 infection that led to an ITP picture [5]. It is unclear if these antibodies act in the same way after COVID-19 vaccination. A better understanding of ITP as related to COVID-19 vaccination will likely emerge as the vaccine is more widely utilized.

The case described herein is, to the authors’ knowledge, the first published report of secondary ITP following immunization with the Moderna COVID-19 vaccine. In reviewing the FDA’s full emergency use authorizations for both Pfizer and Moderna COVID-19 vaccines in prevention of COVID-19 for children six months through four years of age, no adverse bleeding events were listed in clinical trials or post-authorization experience [6,7]. However, it is important to note that early studies and early post-authorization experience may miss rare events such as myocarditis or ITP, where the number of occurrences is below the threshold of detection by these studies (events that occur in the 1/100,000 rate or higher). Because these events require larger populations exposed to the vaccine to become statistically significant, it will be important to follow case reports to help guide providers in management both pre- and post-vaccine administration.

Both Moderna and Pfizer-BioNTech COVID-19 vaccines for individuals six months through four years of age have been available under emergency use authorization since June 2022, and adverse events have been submitted to the Centers for Disease Control and Prevention Vaccine Adverse Events Reporting System (VAERS) by healthcare providers and consumers. Reviewing VAERS showed 15 submissions of “petechiae” in children five years old and younger after mRNA vaccination [8]. However, several submissions suggest alternate causes, such as petechiae occurring minutes after vaccination or a vasculitis occurring around the time of vaccination. Three submissions include the diagnosis of ITP with thrombocytopenia or low platelet count occurring weeks after COVID-19 vaccination [8].

Regarding our case, secondary ITP following viral illness such as enterovirus is certainly a possibility, especially with the history of non-bloody diarrhea presenting in the days after vaccination. However, the clinical presentation is suggestive of secondary ITP after COVID-19 vaccination given the occurrence of acute isolated thrombocytopenia within two weeks of immunization, correspondence with peak age incidence for ITP, lack of clear specific viral syndrome, no further bruising or bleeding, and lack of additional symptoms. In terms of management, our patient was observed without initiation of treatment, as he had no bleeding or new bruising. In the American Society of Hematology 2011 evidence-based practice guideline for immune thrombocytopenia, children with no bleeding or mild bleeding (defined as skin manifestations only) can be managed with observation alone regardless of platelet count [9].

## Conclusions

As more children under five years of age are vaccinated for COVID-19, providers should be made aware of complications, including secondary ITP, given its relatively high incidence in this age group. This case may help provide awareness for physicians, with a potential need for further medical management to prevent serious bleeding events. However, this case alone does not prove causality but provides more awareness toward complications and the need for further surveillance and reporting. Additionally, the decision to offer subsequent COVID-19 vaccination after secondary ITP from initial vaccination depends on individual risk assessment and should be made in consultation with a hematologist, which could include guidance on laboratory monitoring before and after vaccination. Increased awareness by providers will also help with identification and reporting of any future events to VAERS and demonstrate a need for larger studies to determine the true incidence. In turn, this may support future investigations of potential risk factors for developing ITP after COVID-19 vaccination.
